# Pragmatic Quasi-Experimental Controlled Trial Evaluating the Outcomes of Blended CBT Compared to Face-to-Face CBT and Treatment as Usual for Adolescents with Depressive Disorders

**DOI:** 10.3390/ijerph18063102

**Published:** 2021-03-17

**Authors:** Sanne P.A. Rasing, Yvonne A.J. Stikkelbroek, Wouter den Hollander, Heleen Riper, Maja Deković, Maaike H. Nauta, Daan H.M. Creemers, Marianne C.P. Immink, Mariken Spuij, Denise H.M. Bodden

**Affiliations:** 1 Clinical Child and Family Studies, Utrecht University, 3508 TC Utrecht, The Netherlands; y.stikkelbroek@uu.nl (Y.A.J.S.); m.dekovic@uu.nl (M.D.); m.spuij@uu.nl (M.S.); d.bodden@uu.nl (D.H.M.B.); 2 Child and Adolescent Psychiatry, GGZ Oost Brabant, 5427 EM Boekel, The Netherlands; d.creemers@ggzoostbrabant.nl; 3 Trimbos Institute, 3521 VS Utrecht, The Netherlands; whollander@trimbos.nl; 4 Department of Clinical, Neuro- and Developmental Psychology, VU University, 1081 HV Amsterdam, The Netherlands; h.riper@vu.nl; 5 Department of Psychiatry, VU University Medical Centre, 1081 HV Amsterdam, The Netherlands; 6 APH Institute for Health and Care Research, VU University Medical Centre, 1081 HV Amsterdam, The Netherlands; 7 Department of Clinical Psychology and Experimental Psychopathology, University of Groningen, 9712 CP Groningen, The Netherlands; m.h.nauta@rug.nl; 8 Accare Child and Adolescent Psychiatry, Groningen University Centre, 9712 CP Groningen, The Netherlands; 9 Behavioural Science Institute, Radboud University, 6525 XZ Nijmegen, The Netherlands; 10 Triversum, GGZ Noord-Holland-Noord, 1817 EZ Alkmaar, The Netherlands; m.immink@ggz-nhn.nl; 11 TOPP-Zorg, Driebergen-Rijsenburg, 3972 WG Driebergen-Rijsenburg, The Netherlands; 12 Child and Youth Psychiatry, Altrecht, 3524 SH Utrecht, The Netherlands

**Keywords:** adolescents, depression, treatment, blended, CBT

## Abstract

Depression is a major problem in youth mental health. Current treatment is on average effective, but adolescents are hesitant to seek help. Blended treatment could lower the barriers to seeking treatment. Evidence on effectiveness is, however, scarce. The present pragmatic quasi-experimental controlled trial aimed to compare the outcomes of blended cognitive behavioral therapy (CBT) to face-to-face CBT and treatment as usual. A total of 129 adolescents with clinical depression (82.2% female), aged 13–22 (*M* = 16.60, *SD* = 2.03) received blended CBT, face-to-face CBT or treatment as usual. Clinical diagnosis, depressive symptoms, and secondary outcomes were assessed at baseline, post-intervention, and six-months follow-up. Participants receiving blended CBT were, compared to participants receiving face-to-face CBT and treatment as usual, evenly likely to be in remission from their depressive disorder at post-intervention and at six-month follow-up. Depressive symptoms decreased significantly over time in all three conditions, and changes were not significantly different between conditions. Other secondary outcomes (suicide risk, internalizing and externalizing symptoms, severity of depression, and global functioning) did not differ between treatment conditions at post-intervention and six-month follow-up. Since there was no evidence for favorable outcomes for face-to-face therapies above blended CBT, blended CBT may also be an effective treatment format in clinical practice.

## 1. Background

Depression has been identified as one of the most prevalent mental health disorders in adolescents and for boys and girls aged 10 to 19 years and is the most important cause of burden and disability [1]. Depressive disorders show a high comorbidity with other mental health disorders [2], have a high risk of recurrence and chronicity [3,4], and are a major risk for suicidal behavior and completed suicide [5]. Furthermore, depression during adolescence is negatively related to social and family functioning [6,7], associated with poor academic and occupational performance [7,8], and related to poor physical and mental health in later life [9,10].

Several meta-analyses have shown that Cognitive Behavioral Therapy (CBT) and Interpersonal Therapy (IPT) are both effective interventions for adolescents with a depressive disorder [11,12,13,14]. There is ample research showing positive effects and both interventions are qualified as first choice treatment for adolescents suffering from depression in several national and international guidelines [15,16]. However, it is also known that effect sizes are on average small to moderate and not all adolescents show improvement and are free of symptoms after receiving treatment [14,17,18]. Importantly, adolescents themselves mentioned lack of time, high costs, lack of available transport, perceived social stigma, and limited treatment resources as the most important reasons for not seeking help [19,20]. Therefore, computerized interventions, such as internet-based CBT, are often mentioned as alternative for face-to-face treatment, because they contribute to increasing dissemination of mental health care by being scalable and can be made widely available [21]. It has low barriers for participation considering that it is free of costs, is flexible, retains anonymity and therefore carries no stigmatization [22,23], but more importantly, it has proven to be effective in reducing depressive symptoms in adolescents [20]. Nevertheless, unguided computerized treatment for depression may not the best strategy for adolescents, since drop-out, increasing symptom severity and suicidal ideation, all common in youth with a depressive disorder, are hard to detect and tackle in unguided computerized treatment in comparison to face-to-face treatment [22]. To prevent drop-out, improve results of online treatment, and monitor possible adverse events, adolescents should be able to connect with mental health professionals [24]. Taken together, merging face-to-face treatment and computerized treatment into an integrated blended treatment would be the optimal combination of elements from both forms of treatment [25,26,27,28].

Blended treatment can be defined as an integrated treatment, containing face-to-face sessions with a mental health professional combined with computerized therapy which patients follow independently [26]. The computerized part of the treatment is delivered through an online, secured, platform, which can be accessed by pc or smart phone. Adolescents independently work through several modules, containing psycho-education and exercises, and therapists deliver feedback and guidance on the platform [25]. The computerized part is combined with face-to-face sessions. According to therapists and patients, the face-to-face sessions are preferably used for getting to know one another, establishing the therapeutic relationship, and preparing to use the computerized part of the program [26,29]. The face-to-face sessions between adolescents and therapists are synchronized in communication, in which blended treatment differs from guided treatment, where contact with therapist is often online (e.g., via chat) or asynchronous. This means that blended treatment contains the benefits of both face-to-face treatment and computerized treatment; it serves the purpose of being flexible in location and intensity and meets the requirements of personal contact, adaptability in content, and safety in monitoring changes in severity, and according to therapists, it increases the engagement of adolescents in the treatment and enhances self-management [26,30].

Although blended treatment has the potential to be used on a large scale in youth mental health care, the evidence of effectiveness is scarce. To our knowledge, only a few studies have examined the effectiveness of blended treatment for adolescents with a depressive disorder. Two randomized controlled trials compared the effects of face-to-face CBT, computerized CBT, and face-to-face CBT and computerized CBT in conjunction (i.e., blended treatment) to a control condition without treatment among students with mild to moderate depressive symptoms [31,32]. These studies revealed that both blended CBT and face-to-face CBT were effective in reducing depressive symptoms and participants in both conditions showed a larger decrease in depressive symptoms compared to participants in the computerized CBT or control condition. In a third randomized controlled trial, blended CBT was compared to treatment as usual in treating adolescents with depressive disorders [33]. Adolescents in both treatment conditions showed significant reductions in depressive symptoms, with no significant differences between conditions. Two randomized controlled trials by Topooco et al. [34,35] showed that an online program including eight weekly chat-sessions resulted in significantly lower depressive symptoms when compared to control conditions comprising active monitoring or non-specific counseling. Although these findings provide a positive perspective on blended CBT, it is unknown whether not seeing patients face-to-face regularly increases the risk of adverse events including suicide risk [36], and whether the presented effects can be translated to adolescents treated in routine care.

The purpose of the present study was to evaluate the outcomes of blended CBT; the design was described in a study protocol [37], and the study compares the outcomes of Doepressie Blended with Doepressie face-to-face (CBT) and with treatment as usual (TAU). Patients in the latter two conditions had already participated in a previous randomized controlled trial (RCT) comparing face-to-face CBT with TAU [38,39]. The main aim was to evaluate and compare the remission rate of depressive disorders between blended CBT and face-to-face CBT and between blended CBT and treatment as usual in adolescents with clinical depression within routine care. Based on previous research in recent years, we hypothesized that the remission rate for depressive disorders was not different from face-to-face CBT, but was higher compared to treatment as usual. The second aim was to explore the differences in outcomes between blended CBT and face-to-face CBT and between blended CBT and treatment as usual on secondary outcomes: depressive symptoms, suicide risk, internalizing and externalizing symptoms, severity of depression, and global functioning.

## 2. Methods

### 2.1. Ethics

Both studies were approved by the Medical Research Ethics Committee METC Utrecht, The Netherlands (protocol NL61804.041.17 approved on 10 October 2017 and protocol NL34064.041.10 approved on 14 June 2011) and were registered in the Dutch Trial Register (NTR; Trial IDs: NTR6759 registered on 16 October 2017 and NTR2676 registered on 3 January 2011). All participants, and if under the age of 16 years also their parents, provided written informed consent. Results are described according to the CONSORT 2010 statement [40,41] and SPIRIT guidelines [42].

### 2.2. Design and Procedure

A pragmatic quasi-experimental controlled design was used. Data, collected between November 2017 and December 2019, from participants receiving blended CBT [37] was compared to data, collected between December 2011 and June 2014, from participants receiving face-to-face CBT or TAU (in a previously conducted RCT) [38,39]. A detailed description of the study design can be found in the published study protocol [37].

**Figure 1:**
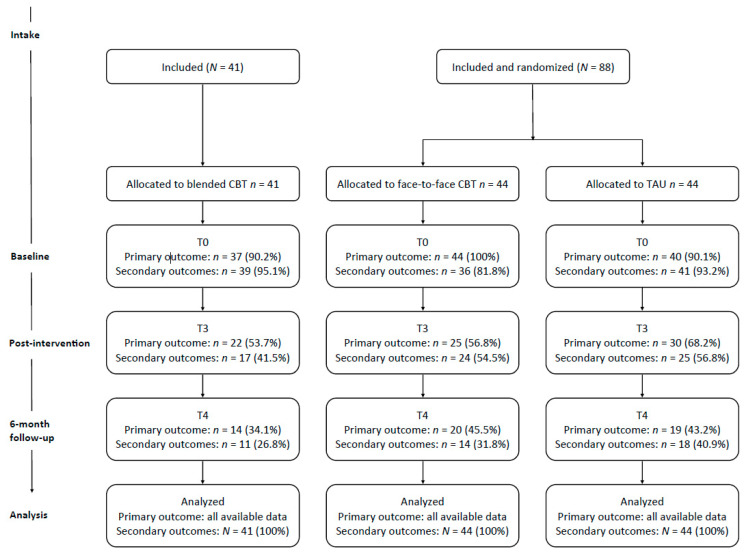
Flow diagram of participants.

Adolescents with a depressive disorder who were referred for treatment in mental health care were informed about the study together with their parents, and they were asked to participate. If the adolescent was 16 years or older, parents would only be approached with the adolescent’s permission. Inclusion criteria were (1) having a clinical diagnosis of a depressive disorder (Major Depressive Disorder (MDD) or Dysthymic disorder), (2) aged 12–21 years, and (3) referred to one of the participating mental health institutions. Exclusion criteria were (1) acute risk of suicide, (2) drug abuse disorder (as primary diagnosis), (3) pervasive developmental disorder (as primary diagnosis), (4) bipolar disorder (as primary diagnosis), (5) day care or admission to the clinical setting, and (6) insufficient knowledge of the Dutch language. Written informed consent was obtained. In both studies, treatment was provided during 15 weeks and could be prolonged to 20 weeks when intermitted by holidays or illness. Assessments were conducted at baseline (T0), during the intervention after five weeks (T1), during the intervention after ten weeks (T2), at post-intervention (T3), and at six months (T4) follow-up. Assessments T0, T3, and T4 are reported in this article. The flow of participants through each phase of the study is shown in Figure 1.

### 2.3. Sample Size

Based on previous research, the within-subject effect sizes of both blended and face-to-face CBT were estimated to be moderate, i.e., blended CBT *d* = 0.76 [43] and face-to-face CBT *d* = 0.53 [14]. To detect a difference in depression diagnoses between the conditions (assuming alpha of 0.05 with a power (1−β) of 0.80, and dropout of 20%), 70 adolescents per condition were required.

### 2.4. Participants

A total of 129 patients (82.2% female) participated in the study and received either blended CBT (*n* = 41), face-to-face CBT (*n* = 44), or treatment as usual (*n* = 44). The adolescents were aged 13–22 years (*M* = 16.60, *SD* = 2.03) when they started treatment. Two participants were 21 when they gave informed consent and turned 22 before treatment started; they remained included. Educational levels were 2.3% lower educational level, 38.4% moderate educational level, and 58.5% higher educational level. Most participants were of Dutch origin (96.4%).

### 2.5. Treatment Allocation

Adolescents participating in the first study were randomly assigned to either face-to-face CBT or treatment as usual [39]. Adolescents participating in the second study all received blended CBT.

### 2.6. Interventions

The TAU condition consisted of a range of different treatments. In this study, mental health institutions offered Interpersonal Therapy (IPT), family therapy, parent counseling, anti-depressant medication, mindfulness training, acceptance commitment therapy (ACT), short-term psychodynamic therapy, (nondirective) counseling, creative therapy, and running therapy. For the purpose of this study, CBT was not allowed within the TAU condition. More detailed information is described by Stikkelbroek and colleagues [39].

For the face-to-face CBT condition, the Dutch protocolized CBT program “Doepressie” was used [44], which is based on the evidence-based treatment program Coping with Depression course for Adolescents (CWD-A) [45]. The program consists of 15 weekly sessions of 45 min each and contains the following components: psycho-education, setting realistic goals, self-monitoring, activation, improvement of social and communication skills, relaxation skills, cognitive restructuring, role play, problem solving skills and relapse prevention. Exercises during the session and homework are used to generalize new skills into daily practice.

For the blended CBT intervention, the program Doepressie was adapted into a blended version [46] with an interactive online environment including four modules, covering the same components mentioned above. The online content of the program was combined with a flexible number of face-to-face sessions with a therapist (with a minimum of five and maximum of 15 sessions). Duration of the face-to-face sessions was equal to the face-to-face condition, namely 45 min each. Throughout the treatment phase, adolescents and therapist could communicate by means of a chat functionality within the program, email or phone. Parents received, comparable to the face-to-face Doepressie, two face-to-face sessions consisting of psycho-education, information about CBT and suggestions on how to contribute to the treatment.

### 2.7. Therapists

Treatment was provided by 60 therapists (91.7% female; 98.1% of Dutch origin) across 16 sites. Therapists were aged 24–63 years (*M* = 42.38, *SD* = 10.89) and had 2–40 years (*M* = 13.54, *SD* = 9.04) of working experience. Most therapists (95.0%) were licensed psychologists with one or more clinical registrations. Two therapists were in training to become cognitive therapists and one therapist was a psychiatric nurse trained as a cognitive therapist. They each treated between one and six participating adolescents. Therapists in the face-to-face CBT and blended CBT condition received a two-day training in delivering the protocolized treatment by a registered clinical psychologist. Therapists in the treatment as usual condition received instruction not to use CBT. Age and level of experience of therapists were evenly distributed across the treatment conditions.

### 2.8. Measures

#### 2.8.1. Primary Outcome

The presence of the diagnosis of depression was measured by the Kiddie-Schedule for Affective Disorders and Schizophrenia, present and lifetime version (K-SADS-PL) [47,48], which was conducted by a trained research assistant. This semi-structured diagnostic interview assesses present and life-time diagnoses and their severity and takes the view of adolescents and parents into account. Test-retest reliability is excellent for present and lifetime diagnoses of major depression and interrater agreement is high (93–100%) [47].

#### 2.8.2. Secondary Outcomes

Depressive symptoms were measured using the Dutch version of the self-report measure Child Depression Inventory-2 (CDI-2) [49,50]. The questionnaire contains 28 items, each consisting of three statements rated from 0 to 2.

Suicide risk was assessed with the self-report questionnaire Suicide Risk Taxation (SRT), consisting of six items rated on a three-point scale. The questionnaire is based on the Suicide Ideation Questionnaire-Jr [51] and on the Suicide Severity Rating Scale [52]. The questionnaire assesses frequency of suicidal thoughts, wishes, plans and actions over the past two weeks.

Internalizing and externalizing symptoms were measured using the Youth Self Report scale (YSR) [53,54] rated by adolescents. The questionnaire assesses a wide range of symptoms with 69 items on a three-point scale, of which 31 items are used for the internalizing symptoms subscale and 32 for the externalizing symptoms subscale.

Severity of depression was assessed with the Clinical Global Impression-Severity scale (CGI-S) [55]. The questionnaire assesses the clinical status of the adolescent, rated by the therapist, on a 7-point scale from ‘normal, no depressive symptoms to ‘most severe depressive symptoms’.

Global functioning of the adolescent was assessed with the therapist-rated Children Global Assessment Scale (CGAS) [56,57]. The ratings of the measure are based on a 0–100 point scale providing an overall estimation of current functioning, taking all available information into account. More impairment in global functioning is represented by lower scores.

### 2.9. Missing Data

An intention-to-treat design was applied and multiple imputation was used to handle the missing data [58] in data measured with questionnaires. The proportions of missing data is presented in Figure 1. Missing data from the clinical interview K-SADS was not imputed. Ten datasets were constructed using the R [59] package Mice [60] (25 iterations) by predictive mean matching. Statistical analyses were performed on each imputed dataset and subsequently pooled using Rubin’s rules [58]. Imputation performance was assessed by comparing the pooled results from analyses performed on the imputed datasets to those performed on the non-imputed data. Sensitivity analyses were conducted to test the robustness of the findings.

### 2.10. Statistical Analysis

Between-group differences in baseline demographic, clinical diagnoses, and clinical characteristics were analyzed using *t*-tests and Mann-Whitney tests for continuous variables and chi-square tests for categorical variables. Odds ratios (OR) for drop-out between participants in the blended CBT and face-to-face CBT condition and between the blended CBT and treatment as usual condition were calculated.

Differences in the primary outcome remission between treatment conditions blended CBT and face-to-face CBT and between blended CBT and TAU were analyzed separately for post-intervention and six-month follow-up, with binomial logistic regression models controlling for age and gender.

To examine secondary outcomes, we analyzed between-group differences in depressive symptoms at post-intervention and at six-month follow-up. Due to non-normal distribution, we used Mann-Whitney tests. We calculated between- and within-group effect sizes (Cohen’s *d*) and their 95% confidence intervals. Next, linear mixed models were constructed using the R package [61] for depressive symptoms, with a random intercept for each participant, while controlling for age and gender. Interaction terms between condition and dummy timepoints (coded binary) were included to test for differences between conditions over time [62]. Mixed models control was used for possible non-independence of the data because of nesting of repeated measurement within individuals, and to deliver unbiased standard errors of the parameter estimates [63]. Additionally, we calculated the participants’ Reliable Change Index (RCI) by dividing the baseline to follow-up difference in depressive symptoms by the standard error of this difference. RCIs larger than −1.96 SDs are qualified as no improvement in symptoms and RCI’s smaller than −1.96 SDs as significant improvement [64]. To test for differences in rates of improvement between groups, binomial logistic regression models controlling for age and gender were fitted with improved RCI as dependent variable.

Next, we analyzed between-group differences in suicide risk, internalizing symptoms, externalizing symptoms, severity of depression and global functioning. Due to non-normal distribution, we used Mann-Whitney tests. Lastly, the same models were fitted as for depressive symptoms as outcome, albeit with suicide risk, internalizing symptoms, externalizing symptoms, severity of depression and global functioning as dependent variable.

## 3. Results

### 3.1. Demographics, Clinical Diagnoses and Clinical Characteristics at Baseline

Participants in the blended CBT did not differ significantly from the participants in the face-to-face CBT or treatment as usual (TAU) condition, respectively, on any of the demographic characteristics age (*t* = 0.41, *p* = 0.68; *t* = 0.79, *p* = 0.43), gender (χ^2^ (1, *n* = 85) = 0.42, *p* = 0.52; χ^2^ (1, *n* = 85) = 0.19, *p* = 0.66), education (χ^2^ (2, *n* = 62) = 2.33, *p* = 0.31; χ^2^ (2, *n* = 69) = 1.29, *p* = 0.52), or ethnicity (χ^2^ (1, *n* = 70) = 0.19, *p* = 0.66; χ^2^ (1, *n* = 73) = 0.03, *p* = 0.86).

The presence of clinical diagnoses did not differ between the blended CBT condition and the face-to-face CBT condition or between the blended CBT condition and the TAU condition, with an exception of the presence of social phobia being significantly lower in the blended CBT condition. An overview of the clinical diagnosis is presented in Table 1.

Further, no differences were found in other clinical characteristics, i.e., depressive symptoms, suicide risk, internalizing symptoms, externalizing symptoms, severity of depression, or global functioning, between participants in the blended CBT and face-to-face CBT or TAU condition. Means and between-group differences are presented in Table 2.

### 3.2. Drop-Out

In total, 58 participants of the 129 (45.0%) dropped out of treatment during the study. In the blended CBT condition, 15 (36.6%) participants dropped out before the planned duration of 15 to 20 weeks of treatment, seven because they ended treatment (i.e., four due to decreased severity of depression and two due to lack of motivation) and eight because they needed more intensive treatment (i.e., they received additional medication for PTSD or anxiety or were clinically admitted after a suicide attempt). In the face-to-face CBT condition, 24 (54.5%) participants dropped out; ten because they discontinued treatment (i.e., four due to decreased severity of depression and six due to lack of motivation or no confidence in treatment), ten because they received more intensive treatment (i.e., they received additional EMDR, emotion regulation therapy or medication or were admitted after a suicide attempt), and four did not start treatment at all. Participants receiving blended CBT and participants receiving face-to-face CBT were evenly likely to drop out (*OR* = 0.48, 95% CI (0.20, 1.15)). In the treatment as usual condition, 19 (43.2%) dropped out of treatment, eleven because they ended treatment themselves (i.e., six due to decreased severity of depression and five due to lack of motivation), six because treatment was intensified (i.e., they were transferred to forensic care or were admitted because of increased suicide risk), and two did not start treatment at all. Participants receiving blended CBT and participants receiving treatment as usual did not differ in the likelihood to drop out (*OR* = 0.76, 95% CI (0.32, 1.81)).

### 3.3. Adverse Events

During the intervention and follow-up period, overall five serious adverse events occurred. In the blended CBT condition, two participants attempted suicide during the intervention. They were referred for more intensive treatment and dropped out of the study. In the face-to-face CBT condition also, two participants attempted suicide, one during the intervention and one after ending the intervention. They also dropped out of the study and received more intensive treatment. A third participant in the face-to-face CBT condition died by suicide between the six- and 12-month follow-up measurement. In the treatment as usual condition, no serious adverse events occurred. Participants receiving blended CBT and participants receiving face-to-face CBT were evenly likely to experience an adverse event (*OR* = 0.70, 95% CI (0.11, 4.42)). Odds ratio for adverse between participants receiving blended CBT and participants receiving treatment as usual could not be calculated due to a value of 0 in the TAU condition.

### 3.4. Treatment Dosage

The treatment dosage specifically for the adolescents who completed their blended treatment consisted of an average of 5.77 (*SD* = 3.22; range 0–12) sessions. Additionally, they processed 57.6% (range = 0–98.7%) of the online content. In the face-to-face CBT condition, adolescents received 15.06 (*SD* = 4.05; range 6–27) sessions on average. Adolescents who completed treatment in treatment as usual condition received on average 15.00 (*SD* = 3.74; range = 4–20) sessions.

#### 3.4.1. Primary Outcome

##### Remission

Before start of the treatment, all participants (blended CBT *n* = 37; face-to-face CBT *n* = 44; treatment as usual *n* = 40) met the criteria for a depressive disorder (i.e., MDD or Dysthymic Disorder). Data from eight participants on the diagnostic criteria before treatment was missing; however, they were diagnosed with MDD in the diagnostic process.

At post-intervention, 47 of the 77 participants (61.0%) were in remission from a depressive disorder. Participants who received blended CBT (*n* = 12, 54.5%) were evenly likely to be in remission from the diagnostic criteria of a depressive disorder compared to participants who received face-to-face CBT (*n* = 17, 68.0%) (*OR* = 0.59, 95% CI (0.17, 2.02)). Compared to participants receiving TAU (*n* = 18, 60.0%), participants receiving blended CBT were also evenly likely to be in remission (*OR* = 0.96, 95% CI (0.31, 3.02)). This indicates no association between remission and treatment condition at post-treatment.

At six-month follow-up, 42 of the 53 participants (79.2%) were in remission. Participants in the blended CBT condition (*n* = 10, 71.4%), compared to participants in the face-to-face CBT condition (*n* = 17, 85.0%) (*OR* = 0.48, 95% CI (0.07, 2.82)) and compared to participants in the TAU condition (*n* = 15, 78.9%) (*OR* = 0.91, 95% CI (0.16, 5.11), were evenly likely to be in remission.

#### 3.4.2. Secondary Outcomes

##### Depressive Symptoms

The levels of depressive symptoms at post-intervention and six-months follow-up did not differ between the blended CBT condition and the face-to-face CBT condition, nor between the blended CBT condition and the TAU condition. Between-group effect sizes between blended CBT and face-to-face CBT and blended CBT and TAU showed no difference in effect (Table 2).

Within-group effect size for blended CBT at post-intervention was moderate (*d* = 0.81, 95% CI (0.36, 1.26); cf. face-to-face CBT *d* = 0.86, 95% CI (0.42, 1.30); TAU *d* = 0.57, 95% CI (0.15, 1.00)). At the six-month follow-up, within-group effect size for blended CBT was large (*d* = 1.11, 95% CI (0.64, 1.57); cf. face-to-face CBT (*d* = 0.92, 95% CI (0.48, 1.36); TAU *d* = 1.14, 95% CI (0.69, 1.59)).

##### Change in Depressive Symptoms over Time

Findings showed a significant effect of time on depressive symptoms at post-intervention (*B* = −6.97, *SE* = 1.49, *p* < 0.001). The estimated decline in depressive symptoms in the blended CBT at post-intervention condition was not significantly different from the face-to-face CBT condition (*B* = 2.62, *SE* = 1.93, *p* = 0.17). The estimated decline in depressive symptoms in the blended CBT condition was also not significantly different compared to the TAU condition (*B* = −0.58, *SE* = 1.92, *p* = 0.76).

For the six-month follow-up, again time had a significant effect on depressive symptoms (*B* = −11.4, *SE* = 1.52, *p* < 0.001). The estimated depressive symptoms showed no significantly different decrease in the blended CBT condition compared to the decrease in the face-to-face CBT condition (*B* = 0.38, *SE* = 1.95, *p* = 0.85). The estimated decline in depressive symptoms comparing blended CBT to TAU was also not significantly different (*B* = 1.37, *SE* = 1.95, *p* = 0.48).

##### Reliable Change in Depressive Symptoms

Between baseline and post-intervention, 30.9% of the participants receiving blended CBT showed a reliable change (i.e., clinically relevant decline) in depressive symptoms, compared to 42.5% receiving face-to-face CBT and 20.0% receiving TAU. We found that participants who received blended CBT were evenly likely to show a clinically relevant decline in depressive symptoms compared to participants who received face-to-face CBT (*OR* =1.71, 95% CI (0.56, 5.16)), meaning no association between reliable change and treatment condition at post-intervention. Compared to participants receiving TAU, participants receiving blended CBT were also evenly likely to show a clinically relevant decline (*OR* = 0.52, 95% CI (0.16, 1.71)).

Between baseline and the six-month follow-up, 46.8% of the participants in the blended CBT condition showed a clinically relevant decline in depressive symptoms compared to 46.4% in the face-to-face CBT condition (*OR* = 1.00, 95% CI (0.35, 2.84)) and 40.9% in the TAU condition (*OR* = 0.74, 95% CI (0.19, 2.89)), evenly likely to show a reliable change in symptoms when controlled for gender and age. This means that no association was found between reliable change and condition at the six-month follow-up. Presented percentages are based on pooled results from ten imputed datasets.

##### Other Outcomes

Between-group differences for suicide risk, internalizing symptoms, externalizing symptoms, severity of symptoms, and global functioning at post-intervention were not significantly different between blended CBT and face-to-face CBT, nor between blended CBT and TAU, as presented in Table 2. At the six-month follow-up, between-group differences between blended CBT and face-to-face CBT, and between blended CBT and TAU on suicide risk, internalizing symptoms, and externalizing symptoms were not significantly different (Table 2).

Findings showed a significant effect of time on internalizing symptoms, severity of symptoms, and global functioning at post-intervention, and no effect on suicide risk and externalizing symptoms. The estimations showed no significantly different decrease in the blended CBT condition compared to the decrease in the face-to-face CBT condition, nor in the blended CBT condition compared to the TAU condition (Table 3). We found a significant effect of time on suicide risk, internalizing symptoms, and externalizing symptoms at six-month follow-up. The estimations showed no significant differences in decrease between blended CBT and face-to-face CBT, nor between blended CBT and TAU.

##### Sensitivity Analyses

Completer-only analyses for individual change over time in depressive symptoms, suicide risk, internalizing symptoms, externalizing symptoms, severity of depression, and global functioning showed no differences with the intention-to-treat analyses, with one exception. Completer-only analyses showed that the estimated decline in depressive symptoms at post-intervention was smaller in the blended CBT condition compared to the face-to-face CBT condition (*B* = 4.79, *SE* = 2.39, *p* = 0.045), with a p-value just below the threshold of 0.05.

## 4. Discussion

In the present study, a pragmatic quasi-experimental controlled trial was conducted to evaluate the outcomes of blended CBT. The main aim was to evaluate and compare the remission rate of depressive disorders between blended CBT and face-to-face CBT and between blended CBT and treatment as usual in adolescents with clinical depression within routine care. The second aim was to explore the differences between blended CBT and face-to-face CBT and between blended CBT and treatment as usual on secondary outcomes depressive symptoms, suicide risk, internalizing and externalizing symptoms, severity of depression, and global functioning.

In total, 71 adolescents of the 129 (55.0%) finished treatment according to their treatment protocol, which means 45.0% dropped out. Adolescents receiving blended CBT did not differ in likelihood to drop out from adolescents receiving face-to-face CBT or treatment as usual. The drop-out rate was high, albeit comparable to other studies on the effectiveness of depression treatments (i.e., 50%) [65]. Nevertheless, drop-out in general is a major problem in mental health care and we need to look for possible ways to increase the number of patients who finish their treatment. Overall, five adolescents experienced a serious adverse event, but again with no difference in likelihood between blended CBT and face-to-face CBT or treatment as usual. Therapists worry that in blended treatment it is too difficult to identify changes in symptom severity and suicidality [66] and, obviously, therapists want certainty about this. Our findings revealed a small number of adverse events, but more importantly, these were not different from treatments consisting of only face-to-face sessions.

Findings showed that 61.0% of the adolescents at post-intervention and 79.2% of the adolescents at the six-month follow-up were in remission from a depressive disorder. In line with our hypotheses, findings showed no difference in likelihood to be in remission between blended CBT and face-to-face CBT. In contrast to our hypotheses, findings also showed no difference in likelihood to be in remission between adolescents who received blended CBT and adolescents who received treatment as usual. All three treatment conditions resulted in a significant decrease in depressive symptoms with a large effect size. No difference was found between blended CBT and face-to-face CBT, nor between blended CBT and treatment as usual in decrease in depressive symptoms, suicide risk, internalizing and externalizing symptoms, severity of depression and global functioning. Summarizing, outcomes of blended were not found to be different from to face-to-face CBT and TAU in treating adolescents with a clinical depression within routine mental health care.

We expected that the remission rate of depressive disorders in blended treatment would be higher compared to treatment as usual, but our findings showed no difference in outcome. Our hypothesis, at the start of the study, was based on scarce research on blended treatment. In recent years, a small, but rising number of studies have examined the effectiveness of blended CBT in treating adolescents with a depressive disorder and presented findings comparable to our results. Kobak et al. [33] showed no difference in decrease of depressive symptoms between adolescents receiving blended CBT or treatment as usual. It is also known that blended CBT was significantly more effective in reducing depressive symptoms in adolescents than minimal attention control conditions [34,35]. Despite our findings not being in line with our hypothesis, they are similar to other recent findings.

We also found, in line with our hypothesis, no difference in likelihood to be in remission between blended CBT and face-to-face CBT. Several explanations can be offered to explain the similar effects of blended CBT to face-to-face CBT and treatment as usual. First, blended CBT and face-to-face CBT have similar treatment techniques, with the modality of delivery being different. Similar results were found by Sethi et al. [31,32] where the decrease in depressive symptoms was equal after receiving blended CBT and standard CBT.

Second, our expectation that treatment as usual would be less effective was based on a meta-analysis by Weisz et al. [11], which showed that evidence-based psychotherapies outperformed treatment as usual. However, based on a few individual studies included in the meta-analysis, the authors also mentioned that the difference in effects between evidence-based therapies and treatment as usual decreased when delivered under clinical practice conditions. It needs to be mentioned that the treatment as usual condition also contained evidence-based treatments such as IPT, anti-depressant medication, and running therapy. This might explain that no difference was found between the outcomes of the blended treatment condition and the treatment as usual condition.

Third, one of the aspects contributing to treatment outcomes is therapeutic alliance [67]. An explorative study on the therapeutic relation in adolescents receiving blended treatment for depression showed that therapist-rated alliance was comparable to alliance in face-to-face treatment in previous research [68]. This implies that the modality of treatment might not impact the therapeutic alliance between therapists and patients and that this factor does not seem to affect blended treatment outcome.

### 4.1. Strengths and Limitations

An important strength of this study is that we compared effects of three active treatment conditions in treating adolescents with a depressive disorder, as suggested by Cuijpers [69]. In addition, we examined the effectiveness of the interventions in adolescents with clinical depression referred for treatment, meaning that these findings can be generalized to clinical care [70]. We also need to mention some limitations. First, in the current design, adolescents were not randomized between conditions. Studying the outcomes of blended CBT without control condition is not preferred. Therefore, we used historical control conditions, of which it cannot be ruled out that the samples differed in baseline characteristics, such as the participants wanting to be included in the blended treatment and having different expectations of treatment than those participating in the RCT. However, based on our baseline measurement, we found no differences between participants in the conditions. Second, the sample size of the study is rather small. We included 129 participants while 210 were required, based on the estimated power. This might have contributed to the difference in findings from the intention-to-treat analysis and completers-only analysis of the decrease in depressive symptoms at post-intervention. Importantly, because the completers-only analysis showed a just significant difference, and all other completer-only analyses confirmed the findings of the intention-to-treat analyses, we interpret that this specific finding was influenced by the small sample size. Third, the drop-out rate of participants was high, namely 45.0%, although comparable to other studies on the effectiveness of depression treatments (i.e., 50%) [65]. This resulted in a loss of power. Fourth, our sample is a relatively highly educated and ethnically homogeneous sample, which is often the case in study samples [71]. Besides the sample being highly educated and ethnically homogeneous, patients with acute suicidal thoughts and behavior were excluded because of safety issues, resulting a part of the patients referred to clinical care being excluded. Therefore, these findings have a limitation in generalization to adolescents in regular care.

### 4.2. Clinical Implications

A previous review demonstrated that the use of blended treatment for adolescents with a depressive disorder largely depends on acceptance by therapists as well as by patients [36]. It is likely that this acceptance increases with more insight into the effectiveness and risks of blended CBT. The current study found no difference in outcomes between blended CBT, face-to-face CBT and treatment as usual, and, with this, we contribute to the knowledge on outcomes of blended treatment. Moreover, we could not establish a difference between the number of adolescents who dropped out from treatment. This means that, on average, adolescents will benefit from blended CBT as much as from face-to-face interventions.

Another important unknown is whether blended treatment, i.e., seeing patients face-to-face much less often, increases the risk of adverse events. Therapists worry that it may be too difficult to identify changes in symptom severity and suicidality [66] and, obviously, therapists want certainty about this. Our findings demonstrated a small number of adverse events, but no difference between treatment conditions. This means that the worry of therapists about the safety of their patients is legitimate, but, based on our findings, blended CBT can be a useful addition to effective treatment for adolescents with a depressive disorder, without increasing the risk of an unseen growing symptom severity of suicidal ideation. Nonetheless, it goes without saying that further research is imperative.

### 4.3. Future Research

This study showed that no difference could be observed between blended CBT, face-to-face CBT and treatment as usual in remission rate and in decrease of depressive symptoms. On average, they are effective in treating adolescents with a depressive disorder. Nonetheless, we also found that not all adolescents showed benefit from the current treatments. An important future step in research is to study which individuals benefits from which intervention; that is, gaining more understanding in the use of prognostic and prescriptive variables or characteristics to determine which treatment works best for whom. The use of predictive information to support treatment selection, i.e., personalizing treatment, might improve the individual effectiveness of therapy.

Further, the absence of knowledge on cost-effectiveness of treatment for adolescents with a depressive disorder is a big gap in the research. It is often suggested that blended treatment could reduce costs compared to face-to-face treatment; however, there are no studies to confirm this yet. A previous study showed that therapists experienced an increase in workload with blended care [66], but it has never been studied in the context of effectiveness and costs.

## 5. Conclusions

The present study showed no differences in effects between blended CBT, face-to-face CBT and treatment as usual: all three treatment conditions resulted in fewer diagnoses of depressive disorder and showed a significant decrease in depressive symptoms. Further, we recommend that we should look beyond the mean effects of the interventions and study whether certain individuals would profit more from one of the interventions than from the others. The ability to identify patients who can benefit from targeted therapies might increase the response to treatment. These results might lead to lowering the threshold for using blended treatment for adolescents with a depressive disorder and might lead to the next, and much needed, steps in precision psychiatry.

## Figures and Tables

**Table 1:** Diagnostic sample characteristics.

	Blended Cognitive Behavioral Therapy (CBT) (*n* = 37)	Face-to-Face CBT (*n* = 44)	Treatment as usual (TAU) (*n* = 40)	Blended CBT vs. Face-to-Face CBT		Blended CBT vs. TAU	
	*n* (%)	*n* (%)	*n* (%)	χ^2^	*p*	χ^2^	*p*
Depressive disorder (MDD or Dysthymic disorder)	37 (100%)	44 (100%)	40 (100%)	*		*	
Bipolar disorder	0	0	0	*		*	
Psychotic disorder	0	2 (4.5%)	0	1.72	0.19	*	
Panic disorder	1 (2.7%)	4 (9.1%)	1 (2.5%)	1.42	0.23	0.003	0.96
Social phobia	1 (2.7%)	13 (29.5%)	8 (20.0%)	10.13	0.001	5.57	0.02
Specific phobia	1 (2.7%)	4 (9.1%)	3 (7.5%)	1.42	0.23	0.90	0.34
Separation anxiety	1 (2.7%)	2 (4.5%)	2 (5.0%)	1.91	0.66	0.27	0.60
Generalized Anxiety Disorder	12 (32.4%)	14 (31.8%)	9 (22.5%)	0.003	0.95	0.96	0.33
Post-Traumatic Stress Disorder (PTSD)	1 (2.7%)	3 (6.8%)	3 (7.5%)	0.73	0.39	0.90	0.34
Acute Stress Disorder	0	0	1 (2.5%)	*		0.94	0.33
Obsessive Compulsive Disorder (OCD)	0	0	0	*		*	
Anorexia Nervosa	0	1 (2.3%)	0	0.85	0.36	*	
Bulimia Nervosa	0	1 (2.3%)	0	0.85	0.36	*	
Attention Deficit and Hyperactivity Disorder (ADHD)	5 (13.5%)	6 (13.6%)	5 (12.5%)	0	0.99	0.02	0.90
Conduct Disorder	0	0	0	*		*	
Oppositional Defiant Disorder (ODD)	0	1 (2.3%)	1 (2.5%)	0.85	0.36	0.94	0.33
Tic disorder	1 (2.7%)	0	0	1.20	0.27	1.10	0.30

Note. * χ^2^ was not calculated due to constant values. Missing data from the clinical interviews was not imputed.

**Table 2:** Between-group differences and effect sizes in secondary outcomes.

	Blended CBT	Face-to-Face CBT	TAU	Blended CBT vs. Face-to-Face CBT			Blended CBT vs. TAU		
	*M* (*SD*)	*M* (*SD*)	*M* (*SD*)	*U*	*p*	*d* (95% CI)	*U*	*p*	*d* (95% CI)
Depressive symptoms T0	25.94 (6.07)	26.58 (9.45)	24.73 (7.46)	746.5	0.64	−0.08 (−0.51, 0.35)	707.0	0.37	0.18 (−0.25, 0.60)
Depressive symptoms T3	18.83 (10.85)	16.43 (13.79)	19.03 (11.88)	141.0	0.10	0.19 (−0.23, 0.62)	187.0	0.52	−0.02 (−0.44, 0.41)
Depressive symptoms T4	14.36 (13.46)	14.19 (16.63)	12.61 (13.06)	60.5	0.38	0.01 (−0.41, 0.44)	95.5	0.89	0.13 (−0.29, 0.56)
Suicide risk T0	3.53 (3.21)	4.55 (4.13)	3.66 (3.40)	702.0	0.40	−0.27 (−0.70, 0.15)	672.5	0.94	−0.04 (−0.46, 0.39)
Suicide risk T3	2.93 (4.11)	2.52 (4.06)	3.01 (4.52)	136.5	0.15	0.10 (−0.33, 0.53)	178.5	0.57	−0.02 (−0.45, 0.41)
Suicide risk T4	1.50 (4.23)	1.73 (4.24)	0.90 (2.67)	71.0	0.74	−0.05 (−0.48, 0.37)	66.5	0.22	0.17 (−0.25, 0.60)
Internalizing symptoms T0	28.20 (9.03)	29.31 (9.08)	28.60 (9.13)	731.0	0.48	−0.12 (−0.55, 0.30)	800.5	0.82	−0.04 (−0.47, 0.38)
Internalizing symptoms T3	22.32 (13.51)	20.41 (14.98)	22.05 (15.15)	161.0	0.40	0.13 (−0.29, 0.56)	188.5	0.77	0.02 (−0.41, 0.44)
Internalizing symptoms T4	17.86 (19.01)	16.03 (14.95)	17.60 (15.38)	66.0	0.56	0.11 (−0.32, 0.53)	118.0	0.58	0.02 (−0.41, 0.44)
Externalizing symptoms T0	11.01 (7.17)	13.43 (9.85)	12.63 (8.09)	784.0	0.19	−0.28 (−0.71, 0.15)	876.0	0.33	−0.21 (−0.64, 0.21)
Externalizing symptoms T3	9.87 (12.50)	11.55 (10.33)	10.55 (11.34)	247.0	0.13	−0.15 (−0.57, 0.28)	251.5	0.17	−0.06 (−0.48, 0.37)
Externalizing symptoms T4	7.38 (10.21)	7.89 (8.56)	8.18 (9.69)	101.0	0.20	−0.05 (−0.48, 0.37)	135.5	0.19	−0.08 (−0.51, 0.35)
Severity of depression T0	4.16 (1.02)	4.16 (1.01)	3.93 (1.36)	663.0	0.34	0.00 (−0.43, 0.42)	686.5	0.56	0.19 (−0.24, 0.62)
Severity of depression T3	2.72 (1.85)	2.53 (1.87)	2.38 (1.53)	310.0	0.66	0.10 (−0.33, 0.53)	286.0	0.15	0.20 (−0.23, 0.63)
Global functioning T0	50.88 (7.32)	49.67 (10.03)	49.24 (10.08)	740.0	1.00	0.14 (−0.29, 0.56)	613.0	0.35	0.19 (−0.24, 0.61)
Global functioning T3	60.85 (18.52)	62.51 (17.82)	64.15 (14.23)	337.0	0.74	−0.09 (−0.52, 0.33)	381.0	0.47	−0.20 (−0.63, 0.23)

**Table 3:** Linear mixed model results of interaction terms between condition and time, on suicide risk, internalizing symptoms, externalizing symptoms, severity of depression and global functioning.

	Blended CBT vs. Face-to-Face CBT			Blended CBT vs. TAU		
	*B*	*SE*	*p*	*B*	*SE*	*p*
Suicide risk T3	0.68	0.66	0.30	−0.01	0.66	0.97
Suicide risk T4	0.05	0.65	0.94	0.69	0.66	0.29
Internalizing symptoms T3	2.07	2.26	0.36	0.44	2.27	0.85
Internalizing symptoms T4	1.98	2.24	0.38	0.39	2.27	0.86
Externalizing symptoms T3	−0.94	1.57	0.55	−0.21	1.56	0.89
Externalizing symptoms T4	0.26	1.56	0.87	−0.35	1.57	0.82
Severity of depression T3	0.19	0.29	0.52	0.32	0.30	0.28
Global functioning T3	−1.91	2.60	0.46	−3.69	2.58	0.15

Note. All results originate from separate multivariate regression analyses, from which the independent variable of interest is here reported.

## Data Availability

The data for the current study is not publicly available due to them containing information that could compromise research participant privacy, but they are available from the corresponding author upon reasonable request.

## Abbreviations

ACT: Acceptance Commitment Therapy (ACT); ADHD: Attention Deficit and Hyperactivity Disorder; CBT: Cognitive Behavioral Therapy; CONSORT: Consolidated Standards of Reporting Trials; CDI-2: Child Depression Inventory-2; CGAS: Children Global Assessment Scale; CGI-S: Clinical Global Impression-severity scale; CWD-A: Coping with Depression course for Adolescents; IPT: Interpersonal Therapy; K-SADS-PL: Kiddie-Schedule for Affective Disorders and Schizophrenia, present and lifetime version; MDD: Major Depressive Disorder; NTR: Nederlands (Dutch) Trial Register; OCD: Obsessive Compulsive Disorder; ODD: Oppositional Defiant Disorder; OR: Odds Ratio; PMM: Predictive Mean Matching; PTSD: Post Traumatic Stress Disorder; RCI: Reliable Change Index; RCT: Randomized Controlled Trial; SPIRIT: Standard Protocol Items: Recommendations for Interventional Trials; SRT: Suicide Risk Taxation; TAU: Treatment as usual; YSR: Youth Self Report scale.
